# Automated Authorship Attribution Using Advanced Signal Classification Techniques

**DOI:** 10.1371/journal.pone.0054998

**Published:** 2013-02-20

**Authors:** Maryam Ebrahimpour, Tālis J. Putniņš, Matthew J. Berryman, Andrew Allison, Brian W.-H. Ng, Derek Abbott

**Affiliations:** 1 School of Electrical and Electronic Engineering, The University of Adelaide, Adelaide, South Australia, Australia; 2 Stockholm School of Economics in Riga, Riga, Latvia; 3 University of Technology Sydney, Sydney, New South Wales, Australia; 4 SMART Infrastructure Facility, University of Wollongong, Wollongong, New South Wales, Australia; National Research & Technology Council, Argentina

## Abstract

In this paper, we develop two automated authorship attribution schemes, one based on Multiple Discriminant Analysis (MDA) and the other based on a Support Vector Machine (SVM). The classification features we exploit are based on word frequencies in the text. We adopt an approach of preprocessing each text by stripping it of all characters except a-z and space. This is in order to increase the portability of the software to different types of texts. We test the methodology on a corpus of undisputed English texts, and use leave-one-out cross validation to demonstrate classification accuracies in excess of 90%. We further test our methods on the *Federalist Papers*, which have a partly disputed authorship and a fair degree of scholarly consensus. And finally, we apply our methodology to the question of the authorship of the *Letter to the Hebrews* by comparing it against a number of original Greek texts of known authorship. These tests identify where some of the limitations lie, motivating a number of open questions for future work. An open source implementation of our methodology is freely available for use at https://github.com/matthewberryman/author-detection.

## Introduction

The field of *data mining* is concerned with the extraction of unknown information and patterns using statistics, machine learning, and artificial intelligence on large scale data sets. Its application ranges from database searches to DNA analysis and text classification [1], [2].

Author attribution is the problem of identifying the authorship of given texts based on characteristics that are not known to the authors themselves. These characteristics are considered reliable because they are inaccessible to conscious manipulation and consistent – under the assumption that a given author has not acquired a mental disorder, such as Alzheimer's disease, where it is known to affect style [1], [3]. Author attribution is also based on the assumption that each author has his/her own writing style that acts as a *fingerprint*, and this is made possible as various measurable features in written text have been shown to be unchanged across a given author's range of writing genres over time [4]–[6]. In 1851, the mathematician Augustus de Morgan tried to determine the authorship of the *Letter to the Hebrews*, in the *New Testament*, by measuring word lengths. Since de Morgan's seminal work, many other methods have been developed [7]–[9]. In 1964, the first computer-assisted studies – as opposed to manual based methods – were performed by Mosteller and Wallace to investigate the authorship of the *Federalist Papers* [10]. Today rapid advances in machine learning, statistical, and software methods have led to computer-based automated systems for detection of authorship [11].

A key problem is to find features in written text that can be quantified in order to reflect an author's style. Once this is achieved, statistical or machine learning techniques can be used to analyse the similarity between pieces of texts. The fast growing areas of machine learning and statistical methods assist in processing the voluminous data, where traditional methods fail due to sparse and noisy data [12], [13].

In recent years, due to an increase in the amount of data in various forms including emails, blogs, messages on the internet and SMS, the problem of author attribution has received more attention. In addition to its traditional application for shedding light on the authorship of disputed texts in the classical literature, new applications have arisen such as plagiarism detection, web searching, spam email detection, and finding the authors of disputed or anonymous documents in forensics against cyber crime [14], [15]. Our focus, here, is the classical literature, and future work may be able to extend our methods to contemporary applications.

This paper is organized as follows. In the Methods section, the discriminant features that are utilized are discussed. Our classification approach, compares the use of Multiple Discriminant Analysis (MDA) with Support Vector Machines (SVM) [16]. These methods are thus briefly introduced. The effectiveness of our methods is investigated by applying them to a benchmark comprised of a known English corpus. Next we apply our methods to the disputed texts of the *Federalist Papers*, as this is a question that has been previously extensively studied. Finally, we revisit de Morgan's problem by applying our methods to the question of authorship of the *Letter to the Hebrews* in the *New Testament*.

## Methods

Generally, there are three types of style markers for authorship attribution: lexical, syntactical, and structural features. Lexical features, for example, include the frequencies of words and letters. Syntactic features include punctuation and grammatically distinct classes of words, such as, articles and prepositions. Structural features use the overall organization of the whole text, such as the length or number of sentences and paragraphs. Since lexical features are easy to extract and the result is usually unambiguous, they play the most important role in computational stylometry [17]–[19].

A number of methods, in the literature, utilize several features and attempt to find the best subset via a feature selection algorithm, leading to accuracies of up to 99%. However this feature selection procedure may be corpus-dependent, thereby limiting applicability for general use [11].

The stylometry marker used in this study is a lexical feature: the frequency of key words. This is one of the best features discriminating between different authors [11], [20]. It is based on the occurrence of a series of non-contextual words such as articles and pronouns, for example ‘the’, ‘and’, ‘of’ in English. This category of words has little or no dependence on the topic or genre of the texts and the technique can easily be applied to different languages – thus it can be argued that these are useful classification features for different texts in order to determine authorship. A tool is needed to break the texts into tokens to then count and choose the most frequently occurring ones [21].

For a given authorship attribution problem, usually there is a group of candidate authors with an associated set of known authorship texts and there is a set of disputed texts requiring classification. Therefore, the data are divided into a training dataset and a disputed dataset. In order to find the set of function words, first, by means of a C++ software program, the number of occurrences of all words in the total dataset (i.e. training dataset plus disputed dataset) is counted. Next, these words are ranked from the most common to the least common, and the first 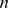 words are chosen, where 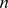 is a parameter of the classification algorithm. We shall call this set of words *function words*. Then the number of occurrences of each function word in each text is counted. For each text, the feature extraction algorithm outputs a vector containing the frequency of occurrences of the function words. This vector is normalized by dividing it by the total word count of the corresponding text, in order to remove the influence of different overall text sizes. The normalized vector is fed into to the classifier as the input.

We examine two powerful supervised learning approaches for performing data classification, Multiple Discriminant Analysis (MDA) and the Support Vector Machine (SVM). The same training dataset is input into both of them. To measure the accuracy of the methods, leave-one-out cross-validation (LOO-CV) is employed.

### Multiple Discriminant Analysis

Multiple Discriminant Analysis (MDA) is a statistical technique designed to assign unknown cases to a known group by using predictor variables. The first step in this technique is to determine whether groups are significantly different with regards to the means of the predictor variables. If there are significant differences, then the variables can be used as discriminating variables. By using discriminating variables, MDA generates discriminant functions that minimize the training error, while maximizing the margin separating the data classes. The basic idea is to form the most possible distinct groups by maximizing the intergroup variance, while minimizing the pooled intragroup variance. If there are *n* groups in a training dataset, 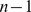 discriminant functions are generated. The 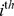 discriminant function is given by:
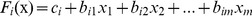
(1)where 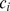 is the constant term, 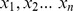 are the observed values of the style markers for each case and 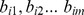 are the corresponding weights of those variables derived from the discriminant analysis [22]–[24].

In this study, we use the SPSS statistical analysis software package [22] to carry out the Multiple Discriminant Analysis. To prevent over-fitting, stepwise MDA is preferred. In stepwise MDA, at each step, all function word counts are evaluated to determine which variables are most effective to the prediction of group membership and those variables are added to the analysis. This process is iterated. It will stop when there is no new variable that contributes significantly to the discrimination between groups. So all the function word counts go into the analysis, but some of them may not contribute toward the discrimination between different authors. So these function word counts do not go into the discriminant function.

Here, MDA utilizes normalized function word frequencies as the discriminant variables and the authors as the grouping variables. The pre-classified training dataset is fed to the MDA and the centroid for each group, that is the mean value of the discriminant function scores, is found. The disputed text is assigned to the author's group that has the smallest Mahalanobis distance between the group's centroid and the disputed text. Mahalanobis distance is calculated by:
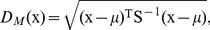
(2)where **x** is the disputed text's vector of discriminant function scores, 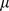 is the mean vector of discriminant function scores for an author's group, **S** is its covariance matrix, and T denotes the matrix transpose.

### Support Vector Machine

The Support Vector Machine (SVM) is a supervised learning algorithm, which uses a training dataset and then classifies the data in question. It classifies data by finding the best hyperplane that separates clusters of features represented in an 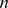-dimensional space. Linear classification SVMs use a real-valued linear function 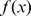, which assigns the 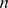-dimensional input vector 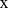 to the positive class if 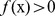, and to the negative class if 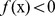. Here 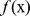 can be written as [16]

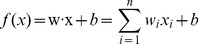
(3)where  denotes the dot product, **w** is the *weight vector* that is the normal vector to the hyperplane and 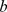 is *bias* or offset of the hyperplane from the origin. Basically a SVM is a two class or binary classifier. When there are more than two groups, the classification problem reduces to several binary classification problems. Multi-class SVMs classify data by finding the best hyperplanes that separate each pair of classes [16].

The geometrical interpretation of a SVM in an 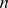-dimensional space is an 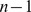 dimensional hyperplane that separates two groups. In this scheme the goal is to maximise the margins between the hyperplane and the two classes. In a more complicated situation, the points cannot be separated by linear functions. In this case, a SVM uses a kernel function to map the data into a higher dimensional space, where a hyperplane is calculated that optimally separates the data. Many different kernels have been developed, however, only a few work well in general. Aside from the linear SVM, common kernels are the polynomial kernel, the Radial Basis Function (RBF) kernel, and the sigmoid kernel as defined here [25], [26]:
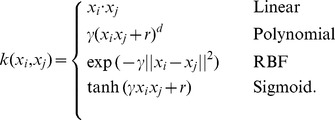



There is no systematic methodology to predict the best kernel with the best parameters for a specific application [24]. In this paper, the best type of kernel and its parameters such as 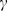 and *r* are found via an optimization procedure that maximizes the accuracy of classification.

### Leave-One-Out Cross-Validation (LOO-CV)

Leave-one-out cross-validation (LOO-CV) is applied to evaluate the accuracy of both methods of classification. At every step, one text is left out from the training dataset and treated as a disputed author text [27]. The classification model is constructed on the remaining data and the algorithm classifies the left out text. The same procedure is applied to all of the training data set and the classification accuracy is calculated by:
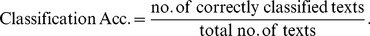
(4)


## Results

We first investigate the performance of both the MDA and SVM methods using a dataset in which authors are known with certainty. For this dataset we use an English corpus of known authors as listed in Table 1. Next we apply our methods to two examples, in order to understand where some of the limitations and open questions lie. First, we examine the question of the disputed texts in the *Federalist Papers* – as we shall see this raises question of what happens when texts possibly are the result of collaboration, and suggests various items for future work. Second, we investigate and revisit de Morgan's author attribution problem of the *New Testament*, where the authorship of the *Letter to the Hebrews* has been debated by scholars since the third century. Here, we use the original *Koine* Greek texts in the *New Treatment*, illustrating how our approach is portable to non-English texts and highlighting a number of limitations for future study.

**Table 1:** English Text Corpus of Known Authorship.

	Bower, B. M. (1871–1940)	5	A Christmas Carol	10	Light of the Western Stars
1	Cabin Fever	6	Dombey and Son	11	The Man of the Forest
2	Casey Ryan	7	George Silverman's Explanation	12	The Mysterious Rider
3	Chip, of the Flying U	8	Going into Society	13	The Rainbow Trail
4	Cow-Country	9	Great Expectations	14	The Redheaded Outfield
5	The Flying U Ranch	10	Hard Times	15	Riders of the Purple Sage
6	The Flying U's Last Stand	11	A House to Let	16	The Rustlers of Pecos County
7	Good Indian	12	Hunted Down	17	The Spirit of the Border
8	The Gringos	13	The Lamplighter	18	Tales of lonely trails
9	The Happy Family	14	Lazy Tour of Two Idle Apprentices	19	To the Last Man
10	The Heritage of the Sioux	15	Little Dorrit	20	The U. P. Trail
11	Her Prairie Knight	16	The Loving Ballad of Lord Bateman	21	Wildfire
12	Jean of the Lazy A	17	Martin Chuzzlewit	22	The Young Forester
13	Lonesome Land	18	Master Humphrey's Clock	23	The Border Legion End
14	The Lonesome Trail and Other Stories	19	A Message from the Sea	24	Light of the Western Stars End
15	The Long Shadow	20	Mrs. Lirriper's Legacy	25	To the Last Man End
16	The Phantom Herd	21	Mugby Junction	26	
17	The Range Dwellers	22	Oliver Twist		**James, Henry (1843-1916)**
18	Rowdy of the Cross L	23	The Holly-Tree	1	The Altar of the Dead
19	Starr, of the Desert	24	A House to Let	2	The Ambassadors
20	The Thunder Bird	25	Hunted Down	3	The American
21	The Trail of the White Mule			4	The Aspern Papers
22	The Uphill Climb		**Doyle, Arthur Conan, Sir (1859-1930)**	5	The Awkward Age
23	Good Indian	1	The Adventure of the Bruce-Partington Plans	6	The Beast in the Jungle
24	The Gringos	2	The Adventure of the Cardboard Box	7	The Beldonald Holbein
25	Good Indian End	3	The Adventure of the Devil's Foot	8	A Bundle of Letters
		4	The Adventure of the Dying Detective	9	The Chaperon
	**Davis, Richard Harding (1864–1916)**	5	The Adventure of the Red Circle	10	Confidence
1	The Amateur	6	The Adventure of Wisteria Lodge	11	The Coxon Fund
2	Billy and the Big Stick	7	The Adventures of Gerard	12	Daisy Miller
3	Captain Macklin	8	The Adventures of Sherlock Holmes	13	The Death of the Lion
4	A Charmed Life	9	Beyond the City	14	The Diary of a Man of Fifty
5	Cinderella And Other Stories	10	The Captain of the Polestar	15	Eugene Pickering
6	The Congo and Coasts of Africa	11	The Doings of Raffles Haw	16	The Europeans
7	The Consul	12	A Duet: A Duologue	17	The Figure in the Carpet
8	The Deserter	13	The Exploits of Brigadier Gerard	18	Glasses
9	The Frame Up	14	The Firm of Girdlestone	19	Greville Fane
10	Gallegher and Other Stories	15	The Green Flag	20	An International Episode
11	In the Fog	16	His Last Bow	21	In the Cage
12	The King's Jackal	17	The Hound of the Baskervilles	22	The Jolly Corner
13	Lion and the Unicorn	18	The Lost World	23	The Lesson of the Master
14	The Log of the Jolly Polly	19	The Mystery of Cloomber	24	Louisa Pallant
15	The Lost House	20	The Parasite	25	Madame De Mauves
16	The Lost Road	21	The Poison Belt	26	The Madonna of the Future
17	The Make-Believe Man	22	Round the Red Lamp		
18	The Man Who Could Not Lose	23	The Sign of the Four		**Lang, Andrew (1844–1912)**
19	The Messengers	24	The Valley of Fear	1	Alfred Tennyson
20	My Buried Treasure	25	The Adventure of the Red Circle	2	Angling Sketches
21	The Nature Faker	26	The Adventure of Wisteria Lodge	3	The Arabian Nights
22	Peace Manoeuvres			4	The Blue Fairy Book
23	The Princess Aline		**Grey, Zane (1872–1939)**	5	The Book of Dreams and Ghosts
24	A Question of Latitude	1	Betty Zane	6	Books and Bookmen
25	Ranson's Folly	2	The Border Legion	7	The Brown Fairy Book
26	The Red Cross Girl	3	The Call of the Canyon	8	Cock Lane and Common-Sense
		4	The Day of the Beast	9	The Crimson Fairy Book
	**Dickens, Charles (1812–1870)**	5	Desert Gold	10	Custom and Myth
1	Barnaby Rudge	6	The Desert of Wheat	11	Grass of Parnassus
2	The Battle of Life	7	Heritage of the Desert	12	The Green Fairy Book
3	Bleak House	8	The Last of the Plainsmen	13	In the Wrong Paradise
4	The Chimes	9	The Last Trail	14	The Library

These are the known texts used in this study for benchmarking the algorithms and indicating their accuracy.

### Benchmark Testing on an English Corpus of Known Authorship

To evaluate the accuracy and reliability of our methods, it is necessary to first test them on a set of texts with known authors, which do not have the limitations and deficiencies of the *New Testament* or *Federalist Papers*. This forms a benchmark for comparing the methods and evaluating the effect of limited text length or training data set size.

**Figure 1:**
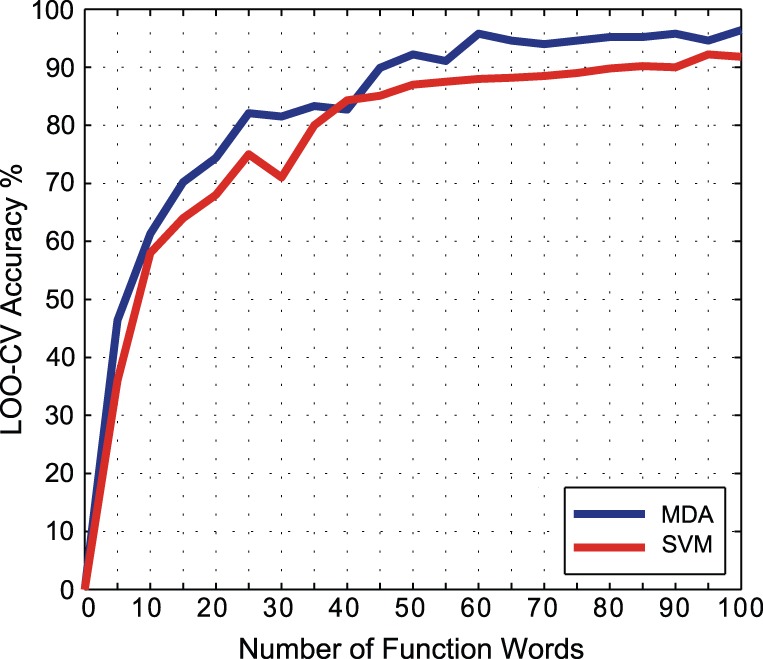
Number of function words vs. LOO-CV accuracy. The SVM uses a polynomial kernel with 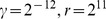, 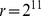 and 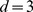. Both MDA and SVM accuracies increase with an increasing number of words up to 100 words, but neither of them improved significantly after this point. These tests use the known English corpus given in Table 1.

Our selected corpus of texts, in English, is obtained from the Project Gutenberg archives [28]. It contains 168 short stories by seven undisputed authors, namely, B. M. Bower, Richard Harding Davis, Charles Dickens, Sir Arthur Conan Doyle, Zane Grey, Henry James, and Andrew Lang. All of these authors wrote fictional literature in English in the same era (late 19th century to early 20th century). So, the genre and the period of time is reasonably uniform and the key discriminant feature is the authors' different styles [23]. Due to the differing lengths of the books, we truncate each of them to approximately the first 5000 words. The texts are listed in Table 1. Both the MDA and SVM classification methods are applied and the results are compared. Figure 1 shows the LOO-CV accuracy for both methods using different numbers of function words. The accuracy of both methods improved with every additional function word up to around 20 function words. Between 20-60 function words, there is still some improvement, but after that the accuracy plateaus.

### MDA Results

Table 2 shows the LOO-CV result of MDA for 7 authors and 100 function words. The numbers in the leading diagonal, show the correct assignments and this occurs in 96.4% of the cases.

**Table 2:** LOO-CV results for MDA classification of the English corpus.

	Predicted Authors							
	Bower	Davis	Dickens	Doyle	Grey	James	Lang	Total
Bower	25	0	0	0	0	0	0	25
Davis	0	26	0	0	0	0	0	26
Dickens	0	0	25	0	0	0	0	25
Doyle	0	1	0	24	0	1	0	26
Grey	0	0	0	1	25	0	0	26
James	0	0	0	0	0	26	0	26
Lang	1	1	0	0	0	1	11	14

Here, 162 out of 168 texts are classified correctly so the accuracy is 96.4%.

### SVM Results

Author attribution problems, with a large number of datasets and several authors, cannot in most cases be resolved with a linear SVM. Choosing the type of kernel and kernel parameters are two significant factors to consider for obtaining the best result. Aside from the number of function words, the kernel's parameters can be optimized to obtain the best classification accuracy. Optimization is carried out to extract the best possible kernel for the given training data. The optimization process is a grid search with exponentially growing sequences of 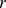 and 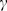, where 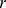 and 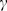 are varied using the values in the following sets: 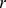 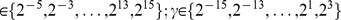. This optimization first employs the LOO-CV technique to check each combination of parameter choices and then selects those parameters that result in the best LOO-CV accuracy. Next, the final model is trained on the whole training set using the chosen parameters [29].

The results are summarised in Table 3. With 95 function words, 92.2% of cases are classified correctly with LOO-CV. This represents an improvement of 12% compared to best results of the recent studies that adopted SVM classifiers [21], [30].

**Table 3:** LOO-CV results for SVM classification of the English corpus.

	Predicted Authors							
	Bower	Davis	Dickens	Doyle	Grey	James	Lang	Total
Bower	22	2	1	0	0	0	0	25
Davis	0	24	0	0	0	2	0	26
Dickens	0	0	24	1	0	0	0	25
Doyle	0	0	0	25	0	0	1	26
Grey	1	0	0	1	24	0	0	26
James	1	0	0	2	0	23	0	26
Leng	0	1	0	0	0	0	13	14

Here, 155 texts out of 168 are classified correctly so the accuracy is 92.2%.

This accuracy is quite good, but here there is a large number of words in each text and the size of training data per author is also large. In many real situations, texts can be rather short and there are few texts per author. Hence, it is necessary to evaluate the affect that limited training data has on the accuracy.

### Affect of Training Dataset Size on MDA and SVM Accuracy

**Figure 2:**
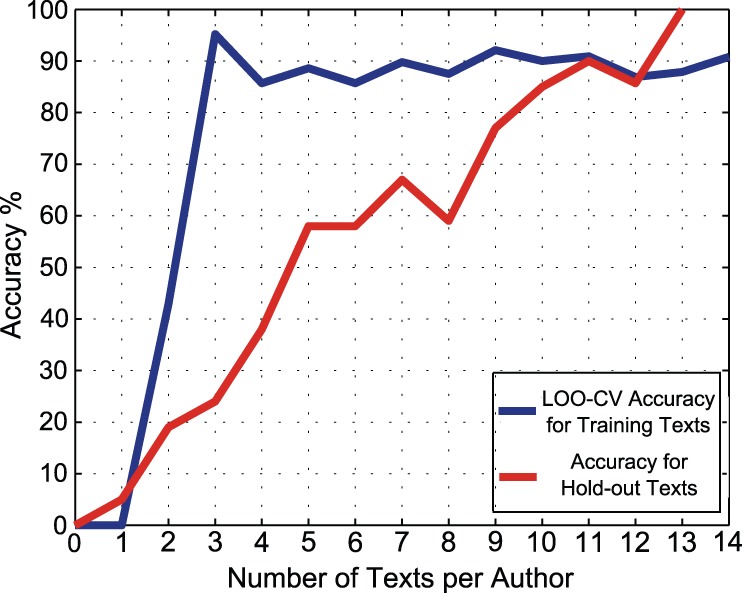
Number of texts per author vs. accuracy of MDA classifier. This graph investigates accuracy versus the size of the training dataset for the MDA case, with a fixed set of 100 function words, for the benchmark English corpus of known texts given in Table 1. The upper curve shows the LOO-CV accuracy of MDA, as a function of the number of author texts, by deliberately limiting the size of the training dataset. The lower curve shows the MDA accuracy that is obtained by inputting the hold-out texts to the classifier at each step.

**Figure 3:**
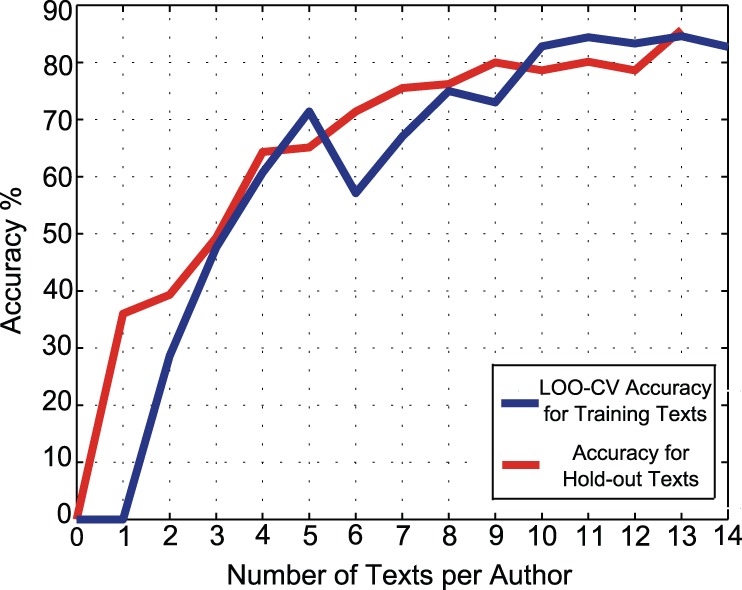
Number of texts per author vs. accuracy of SVM classifier. This graph investigates accuracy versus the size of the training dataset for the SVM case, with a fixed set of 95 function words, for the benchmark English corpus of known texts given in Table 1. The SVM utilizes a polynomial kernel with 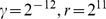 and 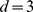.

To investigate the affect of training dataset size, while other variables are kept constant, the number of texts per author is changed. There are different numbers of texts per author available in our dataset. The minimum number of texts per author is 14. Therefore in order to investigate how the dataset size affects accuracy, the classification procedure is repeated with 14 texts per author, then 13 texts per author, and so on, down to zero texts. At each step there are two groups of data, the group of texts that have been used as a training dataset, and the remainder that we call the *hold-out* dataset. As there are two different types of input data (training and hold-out), we can adopt two measures to calculate the accuracy at each step. The first measure is obtained by carrying out LOO-CV across the training dataset. The second method feeds the hold-out texts into the classifiers and it attributes each of the texts to one of the candidate authors. In this test case, we already know the actual authors of texts, so we compare the classifier results with the already-known authorship to find how many of them are correct. The accuracy will be the ratio of the number of correct attributions to the whole number of hold-out texts. Figures 2 and 3 summarize the results for both MDA and the SVM, respectively. In both graphs the accuracies using the training texts and the hold-out texts are shown.

### The Federalist Papers

The *Federalist Papers* are a series of 85 political essays published under the name ‘Publius’ in 1788. At first, the real author(s) were a guarded secret, but scholars now accept that Alexander Hamilton, James Madison, and John Jay are the authors. After a while Hamilton and then Madison provided their own lists declaring the authorship [31], [32]. The difference between these two lists is that there are 12 essays that both Madison and Hamilton claimed individually for themselves. So 73 texts might be considered to have known author(s) while 12 are of disputed authorship. These 12 disputed authorship texts are essay numbers 49–58, 62 and 63. An early study carried out by Mosteller and Wallace (1964) concluded that all of the disputed essays were written by Madison, with the possible exception that essay number 55 might be written by Hamilton [10], [33]. Not all researchers agree with this conclusion. Some scholars also suggest that essay number 64, which is normally attributed to Jay, is written by Madison [31], so we also consider essay number 64 as a disputed text. In total, this gives us 13 disputed essays and 72 undisputed essays. Amongst the undisputed texts, 51 essays are written by Hamilton, 14 essays are written by Madison, and 4 essays are written by Jay. Three essays (numbers 18, 19, and 20) are products of collaboration between Hamilton and Madison [34], [35].

**Figure 4:**
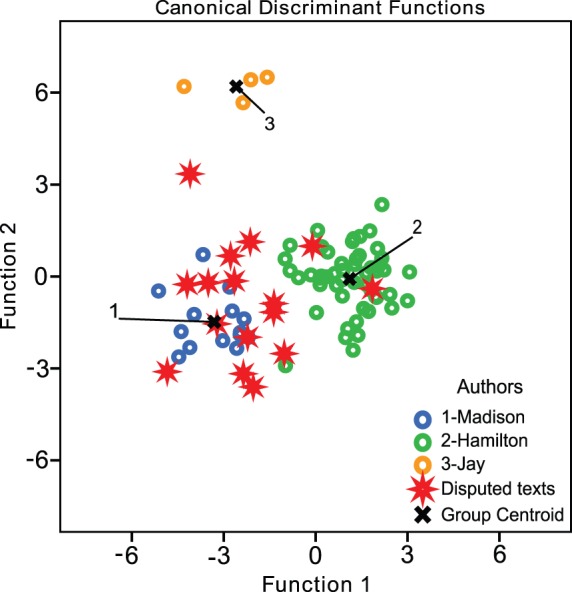
Canonical discriminant functions for the Federalist Papers. This is the result of MDA on the *Federalist Papers* using two discriminant functions. Each point represents a text, which is plotted according to the values of its discriminant functions. Here, 75 function words are utilised, which yields the most accurate result. Open circles indicate known texts, asterisks indicate the 13 disputed texts in question, and the crosses indicate the centroids of the known author clusters.

The texts are obtained from the Project Gutenberg Archives [28]. We put aside the three essays with collaborative authorship and take the remaining 69 essays as the training dataset. The same function word list (see Table 4) is used for our MDA and SVM classifiers. Because there are three authors, MDA produces two discriminant functions, that are shown in Figure 4. For the *Federalist Papers* of undisputed authorship, the LOO-CV accuracy is 97.1%, close to the LOO-CV accuracy for the SVM, 95.6%. In both methods the number of function words required to achieve the highest accuracy is 75 words. The assigned authors for disputed texts for both methods are summarized in Table 5 The MDA results in Table 5 are obtained by attributing each text to the author with the lowest Mahalanobis distance from the text. The Mahalanobis distances are shown in Table 6. A more critical approach is to only select an author based on lowest Mahalanobis distance, if for each contending author the Mahalanobis distance between the text and the contending author's centroid is greater than or equal to the longest distance (LD) between the contending author's known texts and the contending author's centroid. Such cases have a much higher degree of certainty, and are indicated with an asterisk in Table 5.

**Table 4:** Extraction of function words.

rank	English	Federalist	Greek	rank	English	Federalist	Greek
	corpus	Papers	texts		corpus	Papers	texts
1	the	the	kai	41	said	at	loipon
2	and	of	eis	42	when	one	ean
3	of	to	o	43	if	them	hmwn
4	to	and	toy	44	out	people	toyto
5	a	in	na	45	what	these	epi
6	i	a	ton	46	we	if	kata
7	in	be	de	47	been	those	egw
8	he	That	en	48	would	any	ws
9	was	it	to	49	up	most	tas
10	that	Which	thn	50	no	no	tis
11	it	is	pros	51	or	we	legei
12	his	as	ayton	52	man	who	peri
13	you	by	aytoy	53	who	can	alla
14	had	this	dia	54	them	his	meta
15	with	or	den	55	are	must	ostis
16	as	for	ths	56	then	there	panta
17	for	have	h	57	upon	constitution	opoion
18	her	would	twn	58	into	upon	ihsoy
19	at	will	einai	59	their	union	qeos
20	she	not	oti	60	could	such	ymwn
21	but	from	toys	61	your	was	all
22	him	with	oi	62	very	so	hmas
23	not	their	ta	63	little	i	esas
24	is	on	sas	64	do	same	hto
25	on	are	aytoys	65	some	every	xristoy
26	my	government	qeoy	66	like	against	se
27	have	an	mh	67	down	national	omws
28	be	they	moy	68	more	authority	kyrioy
29	me	states	me	69	will	should	qeon
30	they	been	qelei	70	can	our	qelw
31	from	may	dioti	71	over	might	ec
32	this	power	tw	72	did	were	kaqws
33	which	all	ek	73	about	ought	tayta
34	there	other	soy	74	now	into	kyrios
35	one	its	apo	75	see	federal	ypo
36	all	but	aytwn	76	old	general	as
37	so	has	ti	77	only	under	aytos
38	were	state	ihsoys	78	time	public	eme
39	an	more	th	79	know	had	gar
40	by	than	eipe	80	any	shall	seis
81	never	great	aythn	91	come	time	palin
82	before	men	pantes	92	young	well	oy
83	well	only	legw	93	here	united	oytos
84	back	some	pneyma	94	mr	could	di
85	has	less	met	95	made	part	sy
86	other	he	idoy	96	good	us	ihsoyn
87	than	between	chapter	97	eyes	different	epeidh
88	two	each	tois	98	under	members	oyxi
89	how	necessary	para	99	first	particular	yios
90	where	first	men	100	each	legislative	eipen

In this paper, our investigation uses up to 100 function words for the English corpus, the *Federalist Papers*, and the *Koine* Greek texts. The frequencies of function words in each text are used as classification features. The texts are initially pre-processed as follows: (i) all letters are changed to lower case, (ii) all accents are removed, (iii) all ASCII characters not in the set a-z (ASCII codes 97–122) and space (ASCII code 32) are removed without insertion of a space. This is with the exception of a hyphen (ASCII code 45) that is substituted with a space (ASCII code 32), (iv) all headings are removed from the texts, so that they only contain free flowing paragraphs, (v) any extra items added by modern editors, such as editorial notes are removed. Whilst hagiographers wrote the Greek texts in capital letters without accents or punctuation, modern editors insert these items for ease of interpretation. Thus, in order to recover the original Greek text, we apply steps (i) to (v). We do the same to English texts so that they act as a punctuation-free benchmark test. As our software only handles the reduced 27-character ASCII set a-z and space, the Greek text is transliterated using the Table 8. After this pre-processing, all the texts within each corpus are concatenated and word frequencies are counted. Words are ranked in descending order of frequency of occurrence. This is shown in the table below. These key words are called *function words*.

**Table 5:** The predicted authors for the 13 disputed Federalist Papers.

Text No.	MDA	SVM
49	Madison	Madison
50	Hamilton	Madison
51	Madison*	Madison
52	Madison	Madison
53	Madison*	Madison
54	Madison	Madison
55	Hamilton*	Hamilton
56	Madison	Hamilton
57	Madison	Hamilton
58	Madison	Madison
62	Madison*	Madison
63	Madison	Madison
64	Jay	Jay

These results use 75 function words for both methods, which yields the best accuracy. The MDA results are selected using a simplistic approach where the authors with lowest distances (highlighted in bold in Table 6), are selected. However, greater certainty is achieved if the other contending authors have high distances from the text being classified. The cases where both remaining authors have distances greater than or approximately equal to the longest distance (LD) of a known text, are indicated with an asterisk. Thus entries with asterisks have a high degree of certainty, and those without asterisks are less certain and possibly may have resulted from collaboration with the next nearest author.

**Table 6:** Mahalanobis distances from each Federalist Paper of disputed authorship to each author centroid.

Text No.	Mahalanobis Distance to Centroid		
	Madison	Hamilton	Jay
	LD = 2.2	LD = 2.6	LD = 1.7
49	**0.6**	2.0	3.2
50	2.0	**0.8**	2.5
51	**1.1**	3.5	3.9
52	**0.8**	2.3	3.7
53	**0.5**	2.6	2.6
54	**1.3**	1.5	3.5
55	2.9	**0.4**	3.6
56	**1.1**	1.4	3.0
57	**1.1**	1.4	2.9
58	**1.1**	2.2	3.9
62	**0.1**	2.5	3.1
63	**0.9**	2.2	2.2
64	1.9	3.2	**1.4**

The texts with the closest distance to each author centroid are highlighted in bold. The longest distance (LD) between an undisputed authorship text and its author centroid is given in the table header.

Without exception, all asterisked cases are supported by the SVM results in Table 5. Thus we can confirm the conclusion of Mosteller and Wallace [10] that Essay 55 is likely to be by Hamilton and essays 51, 53, and 62 are more likely to be by Madison. We are not able to make a conclusion regarding the remaining essays and suggest that future work investigate the possibility that Essays 49, 50, 52, 54, 56, 57, 58, 63, and 64 might be the result of some degree of collaboration.

The geometry of the MDA method allows us to develop an intuitive new simple method for assigning a likelihood measure to each authorship attribution, which takes into account not only how close a text is to its assigned author centroid but also how far away it is from the second nearest candidate author.

Let us imagine that a disputed text is close to the centroid of Author A, and the next-to-nearest centroid is that of Author B. For high likelihood of a match, we want the ratio 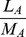 to be as large as possible and certainly greater than unity, where 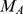 is the Mahalanobis distance between the disputed text and the centroid of Author A and 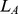 is the longest Mahalanobis distance between Author A's known texts and Author A's centroid. Coupled with this, we want the ratio 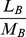 to be as small as possible and certainly less than unity, where 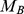 is the Mahalanobis distance between the disputed text and the centroid of Author B and 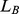 is the longest Mahalanobis distance between Author B's known texts and Author B's centroid. Thus we define the likelihood of a match 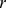 as given by,
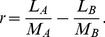
(5)


The certainty of a match increases as 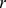 increases and it goes to zero when the two terms are equal, as expected. By applying this methodology to the Mahalanobis distances, in Table 6, we can re-allocate the authorship attribution and rank them according to the likelihood, 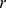, as shown in Table 7.

**Table 7:** Authorship attribution for the *Federalist Papers* ranked by likelihood, 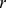.

Likelihood	Author	Essay
*r*		number
20.96	Madison	62
5.74	Hamilton	55
3.40	Madison	53
2.37	Madison	49
2.15	Hamilton	50
1.62	Madison	52
1.26	Madison	63
1.26	Madison	51
0.82	Madison	58
0.14	Madison	56
0.14	Madison	57
0.06	Jay	64
0.04	Hamilton	54

As can be seen in Table 7, there is a relatively high likelihood that Essay 62 was written by Madison. Other assignments have less certainty, and in particular the last seven assignments that have likelihood close to or less than unity are much less certain. How can this be, given the very high accuracy of the MDA method on the English corpus? A likely scenario is that the lower ranked texts are the products of a greater degree of collaboration between the authors, and this remains an open question for future investigation.

### The Letter to the Hebrews

Traditionally the *Letter to the Hebrews* is attributed to the Apostle Paul, also known as Saul of Tarsus. After the third century AD many scholars debated this idea. Three further suggestions for authorship of the *Letter to the Hebrews* are Barnabas, Luke the Evangelist, and Clement of Rome [36]. Luke and Paul are amongst the authors of the *New Testament*, Clement was an apostolic father and Barnabas was an early Christian disciple. Works of these four possible authors with three other *New Testament* authors including Mark, Matthew, John and another apostolic father, Ignatius of Antioch, are tested to determine the most likely author. All of these selected texts are written in the first century. The function word method is used to obtain the set of the stylometry vectors, and both MDA and SVM are used for classification.

The *New Testament* and non-canonical texts are obtained from Society of Biblical Literature [37] and Christian Classical Ethereal Library [38], respectively. All the source texts are in *Koine* Greek and we pre-process them to remove any headings, verse numbers, and punctuation introduced by modern editors. Note that the original *Koine* Greek has no punctuation marks and no accents. As our software handles only the ASCII characters a-z and space, we transliterate the Greek text into our required ASCII set using the look-up table given in Table 8. A limited number of certainly known author texts are available from the first century. The length of text for each author varies from 5,000 words to 50,000 words. Based on our experiments, an equal length of text per author, gives improved accuracy. A possible solution might be to truncate the texts to make them all of equal length, however this is problematic. This is because we have limited data size and need to utilize and extract any information hidden in all the available data. To address this difficulty, the known texts of each author are concatenated together and divided by four. The length of each text for different authors now varies between 1,600 to 10,000 words per text, which reduces the ratio of largest to smallest text from 10 to 6.25. Table 9 lists the names of the texts used for each author along with their word lengths. The vector of the frequency of occurrences of the function words is normalized by dividing by the number of words per text. The normalized vectors are now ready for entry into the classification stage. This method alleviates the problem of different dataset sizes.

**Table 8:** Greek to English character look-up table.

Greek	English equivalent
α	a
β	b
γ	g
δ	d
ε	e
ζ	z
η	h
θ	q
ι	i
κ	k
λ	l
μ	m
ν	n
ξ	x
*o*	o
π	p
ρ	r
σ	s
τ	t
υ	u
φ	f
χ	c
φ	y
ω	w

This look-up table is used to transliterate the *Koine* Greek alphabet to an English equivalent. This is used because the software only handles ASCII characters a-z and space. The software only requires a one-to-one correspondence between Greek letters and our reduced ASCII set, and thus the actual ASCII characters can be entirely arbitrary.

**Table 9:** Source Texts from New Testament and Apostolic Fathers in *Koine* Greek.

	Author	Book	Words
1	Luke	Gospel of Luke	21,318
		Acts of the Apostles	20,612
			
2	Mark	Gospel of Mark	12,844
3	Matthew	Gospel of Matthew	21,957
4	John	Gospel of John	23,887
5	Paul	Epistle to the Romans	8,341
		First Epistle to the Corinthians	7,932
		Second Epistle to the Corinthians	5,149
		Epistle to the Galatians	2,617
		Epistle to the Philippians	1,890
		First Epistle to the Thessalonians	1,666
		Epistle to Philemon	335
6	Clement	First Epistle of Clement	9,833
7	Ignatius	To the Ephesians	1,828
		Letter to the Magnesians	1,053
		Letter to the Trallians	938
		Letter to the Romans	1,092
		Letter to the Philadelphians	965
		Letter to the Smyrnaeans	1,149
		Letter to Polycarp, Bishop of Smyrna	813
8	Barnabas	The Epistle of Barnabas	6,710
9	Disputed	Letter to the Hebrews	5,819

**Figure 5:**
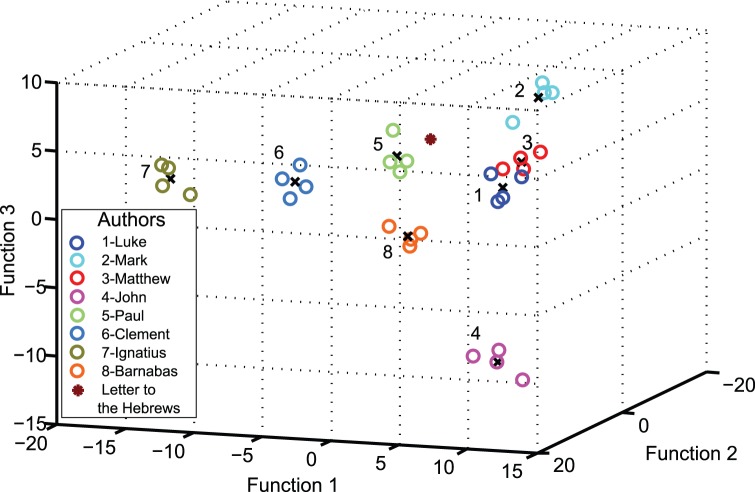
First three canonical discriminant functions for New Testament authors and Apostolic Fathers. This plot shows the MDA results for the Greek texts, in order to determine which author's cluster of texts is closest to the *Letter to the Hebrews*. We use seven discriminant functions in this analysis, however, only the first three discriminant functions are plotted here for illustrative purposes. There are four data points for each author, as all their known texts are concatenated and divided by four.

The table below lists all the Greek texts we used to compare against the *Letter to the Hebrews*. We restrict this training dataset corpus to those texts that are largely undisputed. The listing is grouped according to authorship and the numbers in the right column represent the total word count for each text. In order to reduce the variation of total word count from author to author, we concatenate the texts in each author group and divide by four. This is why, in Figure 5, there are four data points for each author.

Applying stepwise MDA to the training dataset gives an LOO-CV accuracy of 90.6%, which is quite good for such a small dataset with several authors. Figure 5 shows the first three discriminant functions for all texts.

Note that there are seven discriminant functions and the plot shows three of them, for illustrative purposes only. However, in order to calculate actual Mahalanobis distances, we consider all seven functions. Table 10 shows the Mahalanobis distance between the *Letter to the Hebrews* and each of the author centroids. Note that Table 10 also shows the longest Mahalanobis distances of each author's known texts to the respective group centroid. The results show that whilst the *Letter to the Hebrews* is indeed closest to Paul, it is nevertheless further away than all the undisputed texts of Paul. This illustrates the difficulty that underlies the centuries of disagreement between scholars on the authorship of the *Letter to the Hebrews*. The second closest author is Luke, who is also one of the mooted authors. Moreover, using a SVM with an optimized polynomial kernel, an LOO-CV classification accuracy of 87.5% is obtained and the *Letter to Hebrews* is attributed to Luke. In fact, an early statement on the authorship of the *Letter to Hebrews* suggested that Paul initially wrote it in Hebrew and Luke translated it into Greek [39]. So one possible hypothesis is that we are seeing the effect of translation on the style of an author and this is consistent with the results of our analysis.

**Table 10:** The Mahalanobis distance between the *Letter to the Hebrews* and the centroids of authors.

Authors	Mahalanobis	Longest distance
	Distance	(LD)
Luke	12.7	5.9
Mark	16.5	9.2
Matthew	14.0	6.9
John	18.7	10.0
Paul	**11.4**	7.4
Clement	13.5	6.8
Ignatius	22.7	8.1
Barnabas	15.0	9.6

The Mahalanobis distance is calculated using the values of all seven discriminant functions. The first column shows the Mahalanobis distance between the disputed *Letter to the Hebrews* and the centroids of known author texts. The Mahalanobis distance in the second column is the longest distance (LD) between an author centroid and known texts by the same author. If a disputed text is classified outside this LD bound, then it is only a weak match. The lowest Mahalanobis distance is indicated in bold and belongs to the Apostle Paul. However, this is outside the LD bound (i.e. 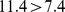) and the likelihood index is less than unity at 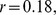 suggesting a weak match.

### Limitations of Study

A key assumption underlying all attempts at automated authorship attribution is that the authors in question write with a consistent style. It is known that style can dramatically change if a mental disorder, such as Alzheimer's disease, is acquired. A limitation, in the specific case of the *Letter to the Hebrews* is the small number of known-authored texts. Could there be other authors in existence that are closer to the *Letter to the Hebrews* than Paul? There are many extra-canonical texts in existence, and future work must exhaustively check these when they become available in electronic format. Whilst the likelihood function we adopted is simple and provides *relative* ranking, it is without characteristic scale and is not appropriate for absolute comparisons from corpus to corpus. Also it implicitly approximates a hyperbolic distribution to the data and assumes the points are spread in a circular symmetric fashion rather than in an ellipsoid. Thus, future work may be carried out in order to further elaborate the likelihood function.

### Future Work

In this study we focussed on stripping the text of any punctuation, as the developed classification methods are then readily portable to other languages such as *Koine* Greek. Thus when we tested our techniques using known English texts, we also stripped the texts of all punctuation to get them into the form of interest. Future work that specifically focusses on the authorship of English texts may benefit from including punctuation, as it possesses style information that may assist in characterizing an author. When extending the work to classify authorship of emails and SMS messages, it may be of greater importance to not only include all natural punctuation but also numbers, emoticons, letter case, redundant spaces, and even idiosyncratic errors. Future work may also investigate different types of feature vectors for classification, other than word frequency, such as word recurrence interval (WRI) [2]. A potential advantage of WRI is that it removes any genre-dependence due to the specific use of words – as it measures how words cluster, whilst disregarding the actual words used.

In regard to elaborating the likelihood weighting for ‘soft’ classification of each text, possible future directions may consider the use least squares optimisation [40]–[42] or fuzzy c-means (FCM) methods [43]–[45].

### Conclusion

In conclusion, we develop a methodology for automated detection of authorship, using the frequency of function words as classification features. There are three critical steps: (i) preprocessing the texts, (ii) extracting classification features, and (iii) performing classification. In regards to the third step, this work compares the performance of a MDA classifier to that of a SVM classifier. Whilst the accuracy of both methods is better than 90%, the SVM is somewhat limiting as it provides only binary decisions. On the other hand the MDA approach allows more flexibility, and enables us to develop a method of ranking authorship attribution according to likelihood. For future work, the MDA approach may therefore be more useful as a method for investigating the degree of collaboration between authors.

With regards to the disputed essays of the *Federalist Papers*, both the MDA and SVM approaches confirm present consensus of scholarship that Essay 62 is indeed written by James Madison. Furthermore the MDA method reveals that the match between Madison and Essay 62 has the highest degree of certainty out of all the 13 disputed essays.

On the question of authorship of the *Letter to the Hebrews*, we find using the MDA method that texts of the Apostle Paul are the closest in style, followed second by Luke the Evangelist. This would appear to favour the traditional belief that Paul is the author. However, the corresponding Mahalanobis distance is longer than the furthest distance between any of Paul's known texts and their stylometric average, suggesting the link between Paul and the *Letter to the Hebrews* is weak.

Thus there are two hypotheses to investigate in future work: (i) could the *Letter to the Hebrews* have been originally written in Hebrew by Paul, and then later translated into Greek by Luke? or, (ii) could there be a further extra-canonical author that is closer in style to the *Letter to the Hebrews*? At present, only a small subset of existing *Koine* Greek texts are available in electronic format, and as further *Koine* texts become available in the future, more exhaustive tests can be carried out.

### Additional Information

#### Software

The LIBSVM library of SVM routines is publicly available [46].
